# Correction: Selective CO_2_ reduction to HCOOH on a Pt/In_2_O_3_/g-C_3_N_4_ multifunctional visible-photocatalyst

**DOI:** 10.1039/d3ra90047a

**Published:** 2023-05-26

**Authors:** Jiehong He, Pin Lv, Jian Zhu, Hexing Li

**Affiliations:** a Education Ministry Key and International Joint Lab of Resource Chemistry, Shanghai Key Laboratory of Rare Earth Functionazl Materials, Department of Chemistry, Shanghai Normal University Shanghai 200234 China hexing-li@shnu.edu.cn +86 21 64322272 +86 21 64322272

## Abstract

Correction for ‘Selective CO_2_ reduction to HCOOH on a Pt/In_2_O_3_/g-C_3_N_4_ multifunctional visible-photocatalyst’ by Jiehong He *et al.*, *RSC Adv.*, 2020, **10**, 22460–22467, https://doi.org/10.1039/D0RA03959D.

In the original manuscript, the authors regret that full details of the photocatalytic CO_2_ reduction at high pressure was not included. An amended Experimental section 2.3 has been included below.

## Section 2.3: Activity test

### Photocatalytic CO_2_ reduction under atmospheric pressure

The photocatalytic CO_2_ reduction was performed in a self-made 50 mL quartz reactor. Before reaction, 20 mg catalyst was dispersed in 10 mL ultra-pure water under ultrasonication for 30 min. Then, 1 mL TEOA (Aladdin) was introduced as a sacrificial agent. The suspension was transferred into a 50 mL quartz reactor with two vents. High purity CO_2_ (99.99%) was introduced into the reactor and bubbled for 0.5 h to evacuate the air inside the reactor. Then the reactor was sealed and filled with CO_2_ to 1 atm. For each run of reactions, the reaction system was irradiated for 4 h by four 3 W LED lights with a wavelength of 420 nm at a constant temperature of 35 °C and stirring rate of 800 rpm. The gas products were analyzed using a gas chromatographer equipped with a HP-PLOT Q column connected with the FID detector and TDX-01 connected with the TCD detector. The liquid products were detected using ion chromatography (Dionex DX-320) with an analytical column (Dionex IonPac AS19-4 μm Analytical Column).

### Photocatalytic CO_2_ reduction under high pressure

The photocatalytic CO_2_ reduction under high pressure was performed in a self-designed autoclave (Shanghai Yanzheng, YZPR-50, China) equipped with light irradiation windows made of sapphire glass. It is a 50 mL stainless steel reactor with a circular light irradiation window on top (∼3 cm thick, 2.5 cm diameter). The reaction unit includes a gas control unit, a booster unit, an autoclave unit, a temperature control unit, and a gas post-treatment unit.

Since the light window was made of sapphire glass, which possessed high light transmissivity and hardness, this resulted in good stability under high pressure. In addition, for experimental safety, the photoreactor is equipped with a rupture disc, and the reaction pressure is factory calibrated, which can withstand 10 MPa of pressure. The light intensity was confirmed at 55 mW cm^−2^ using an irradiatometer (CEAulight, CEL-NP2000, China). Before the reaction, the air in the reactor was replaced with CO_2_ and pressurized to 40 atm. After the reaction, the liquid and gas products were separated with a gas post-treatment unit, which was further analyzed like the reaction under atmospheric pressure.

The scientific conclusions of this article remain unaffected by the changes.

The Royal Society of Chemistry apologises for these errors and any consequent inconvenience to authors and readers.

## Supplementary Material

